# Remote primary care consultations for people living with dementia during the COVID-19 pandemic: experiences of people living with dementia and their carers

**DOI:** 10.3399/BJGP.2020.1094

**Published:** 2021-05-18

**Authors:** Remco Tuijt, Greta Rait, Rachael Frost, Jane Wilcock, Jill Manthorpe, Kate Walters

**Affiliations:** Research Department of Primary Care and Population Health, University College London, London.; Research Department of Primary Care and Population Health, University College London, London.; Research Department of Primary Care and Population Health, University College London, London.; Research Department of Primary Care and Population Health, University College London, London.; The Policy Institute, King’s College London, London.; Research Department of Primary Care and Population Health, University College London, London.

**Keywords:** caregivers, COVID-19, dementia, primary health care, qualitative research, remote consultation, telemedicine

## Abstract

**Background:**

COVID-19 has accelerated remote healthcare provision in primary care, with changes potentially permanent. The implementation of remote provision of health care needs to be informed by vulnerable populations, such as people living with dementia.

**Aim:**

To understand the remote healthcare experiences of patients living with dementia and their family carers during the COVID-19 pandemic.

**Design and setting:**

Qualitative interviews with community-based patients living with dementia and their carers were carried out between May–August 2020, while the COVID-19 pandemic was ongoing in England.

**Method:**

Semi-structured interviews were conducted remotely by telephone or video call with 30 patients living with dementia and 31 carers. Data were analysed using thematic analysis.

**Results:**

Three main themes were derived relating to: proactive care at the onset of COVID-19 restrictions; avoidance of healthcare settings and services; and difficulties with remote healthcare encounters. People living with dementia and their carers felt check-up calls were reassuring but limited in scope and content. Some avoided healthcare services, wishing to minimise COVID-19 risk or reduce NHS burden, or encountering technological barriers. Difficulties in remote consultations included lack of prompts to remember problems, dealing with new emerging difficulties, rescheduling/missed calls, and inclusion of the voice of the person with dementia.

**Conclusion:**

While remote consultations could be effective, proactive calls could be more structured around needs. Consideration should be given to replace non-verbal prompts to describe problems, particularly for new health concerns. In continuing remote consultations, it is important to facilitate engagement with patients living with dementia and their carers to ensure good practice.

## INTRODUCTION

Current projections estimate that, by 2025, there will be over a million people living with dementia in the UK.^1^ Around 60% are living in the community, many supported by family members.^2^ NHS primary care provides continued support for both patients living with dementia and their carers, with guidance recommending regular contact and reviews.^3^^–^^5^

Following the onset of the severe acute respiratory syndrome coronavirus 2 (SARS-CoV-2) pandemic, UK primary care practices changed contacts with their patient populations to reduce transmission of the virus. These changes included telephone and video consultations instead of face-to-face consultations, thereby encouraging remote healthcare encounters as the prime point of contact.^6^^,^^7^ This accelerated the introduction of a variety of remote consultations in primary care, although a 2016 survey found two-thirds of UK practices were offering frequent remote consultation by telephone, albeit with general reluctance to use other technology.^8^

Remote healthcare encounters in primary care focus on sharing biomedical information^9^ and are shorter than face-to-face consultations. Nevertheless, they are satisfactory for both patient and practitioner,^10^ and patients without cognitive impairment have been found to accurately recall the content of such consultations.^11^ For people living with dementia, trials of remote consultations using video technology showed this was a feasible approach to providing ongoing clinical care;^12^ however, this was either precipitated by an in-person visit explaining the remote consultation or conducting other testing, or the provision of additional technology and coaching.^13^^,^^14^ However, digital exclusion among older populations is high compared with that of other age groups,^15^ especially those with a lower income and lower (eHealth) literacy levels.^16^^,^^17^

The UK General Medical Council guidance on remote consultations encourages their use if the clinical need or treatment request is straightforward, the patient can receive all the information they need, there is no need to examine the patient, and the patient has capacity to decide about treatment.^18^ Problems may arise if there are complex clinical needs, difficulties in accessing patient records, or uncertainty regarding the capacity of the patient. For patients living with dementia, there may be symptoms-related complications,^19^^,^^20^ or other illnesses^21^ that could make remote consultations more difficult. There are initial indications, however, that telemedicine can be used to support people living with dementia with disease-specific problems.^22^

**Table:** How this fits in

Primary care consultations in the UK have shifted towards remote methods as a result of the COVID-19 pandemic. This qualitative study explores the experiences of and attitudes towards remote consultation through thematic analysis of interviews with people living with dementia and their carers. COVID-19-related check-up calls were found reassuring, albeit somewhat lacking in practical recommendations. Avoidance of healthcare services for numerous reasons was reported, including COVID-19 risk as well as relieving the strain on the NHS. When individuals did have remote consultations, however, these were mostly by telephone and commonly managed by the carer. Primary care professionals engaging with people living with dementia and their carers may need to make extra effort to ensure the efficacy of remote healthcare consultations with patients living with dementia and their carers.

Understanding the experiences of remote health care for people living with dementia is fundamental to shaping recommendations that will be relevant in times of restricted access to healthcare services, as well as the implementation of remote health care following the COVID-19 pandemic.^23^^,^^24^ The aim of this study was to explore experiences of remote health care during the COVID-19 pandemic in the community, using a qualitative approach to provide insight on how implementation of remote consultations can take account of or compensate for disabilities or other needs related to skills and confidence, including access to technology.

## METHOD

### Design and setting

This study aimed to explore the experiences of post-diagnostic care for people living with dementia and their carers at home. As the impact of COVID-19 and the relevant restrictions on healthcare access became clearer, permissions were received to conduct all interviews remotely and to include COVID-19-related questions. In this study, maximum-variation purposive sampling was used to try to reflect a variety of ages, type of dementia, sex, ethnicity, and rurality. Participants were recruited from primary care practices in North London and Thames Valley, secondary care NHS mental health memory services in East London, and online recruitment (Join Dementia Research^25^ and Alzheimer’s Society Research Network^26^).

### Participant recruitment

Participants were screened using the following inclusion criteria:

- having a diagnosis of dementia and living in the community;
- having capacity to take part in and consent to an interview; and
- sufficient comprehension and grasp of the English language, as interpretation/translation costs were not covered by the funder.

Eligible individuals with a dementia diagnosis were posted or emailed an invitation letter and a study information sheet by the research team, their GP or memory service, and invited to contact the study team to express interest. The study team provided further information on interview procedures and checked potential participants’ understanding of the research aims before scheduling a date and time, and establishing preferences for interview method (video or telephone call). People living with dementia were asked to consider a family member or friend involved in their care and support who could also participate and establish if they would be willing to share the study information (referred to as ‘carers’ in this paper). Interviews were offered individually, but, if both the person living with dementia and their carer preferred, they could be conducted jointly as a dyadic interview. Participants who opted for a dyadic interview were each offered the opportunity to have an individual interview to follow up any further points.

### Data collection

Consent procedures were audio-recorded with permission and discussed with each participant point by point. Interviews were conducted by a trained qualitative researcher with a background in psychology and dementia, and were semi-structured using a topic guide (see Supplementary Appendix S1). This had been developed by the research team, who have a range of experience of qualitative research, family caring, and clinical practice with people with dementia and their carers. Feedback was sought from a former carer and a person living with dementia on the initial and final versions of the topic guides. At the end of the interview, participants were asked demographic information, including postcode data, which were checked against the Office of National Statistics Rural–Urban Classification 2011,^27^ as well as the Index of Multiple Deprivation.^28^ Interviews were conducted May–August 2020, with all being conducted by telephone except for one video interview.

Interviews were audio-recorded and transcribed verbatim by a third-party company, except for six random files that were transcribed by a study author for familiarity and data immersion. Returned transcripts were checked against the audio files for discrepancies and pseudonymised, maintaining references to other participants by use of participant codes. Transcripts were imported into NVivo software version 12 to facilitate analysis.

### Data analysis

Initial coding was done inductively, line by line, and then grouped into themes where codes described similar experiences, a regular method of reflexive thematic analysis.^29^ Two authors individually analysed COVID-19 data from the first 20 interviews and independently developed thematic frameworks. Frameworks had many coding similarities, and different codes and themes were discussed and refined to form a single framework, driven by the data. Analysis of further interviews was done reflexively, adding to existing themes or developing new ones as appropriate. The overall interpretation and meaning were discussed at several meetings and developed with all authors, a multidisciplinary team providing expert insight on primary care, dementia care, social care, ageing, and qualitative research methods.

## RESULTS

Thirty people with dementia and 31 carers participated in a total of 46 interviews (average duration: 27.5 minutes), of which 15 were dyadic interviews. One person with dementia declined to participate on the day of the interview but their carer was interviewed. The final sample (Table 1) was diverse with regards to age, carer relationship, ethnicity, types of dementia, and deprivation.

**Table 1.** Demographics of people living with dementia (*N* = 30) and their carers (*N* = 31)

**People with dementia**		**Carers**	
**Sex, *n***		**Sex, *n***	
Female	17	Female	20
Male	13	Male	11

**Ethnicity, *n***		**Ethnicity, *n***	
White British	19	White British	18
South Asian	4	South Asian	5
Black Caribbean	4	Black Caribbean	4
White Other	3	White Other	4

**Age, years**		**Age, years**	
Range	68–100	Range	45–84
Mean	82	Mean (spouses)	76
		Mean (children)	57

**Age distribution, years**		**Relationship, *n***	
65–74	6	Child	15
75–84	13	Spouse	14
85–100	11	Friend	2

**Dementia diagnosis**			
Alzheimer’s disease	9		
Unspecified	9		
Mixed	7		
Vascular	5		

**Diagnosis year**			
Median	2019		
Range	2011–2019		

**Living status, *n***			
With carer	20		
With other family	4		
Sheltered accommodation^a^	3		
Alone	3		

**Rural–Urban Classification**			
Urban major conurbation	24		
Urban city and town	2		
Rural town and fringe	1		
Undisclosed	3		

**IMD quintile^b^**			
1	3		
2	8		
3	6		
4	4		
5	6		
Undisclosed	3		

a *No care staff on site; often includes adaptations for disability.*

b *1 = most deprived, 5 = least deprived. IMD = Index of Multiple Deprivation.*

### Findings

Interview data were analysed to derive themes related to experiences of remote healthcare encounters. The following three themes were identified:

- proactive care at the onset of COVID-19 restrictions;
- avoidance of healthcare settings and services; and
- difficulties with remote healthcare encounters.

Participants discussed a range of primary and secondary healthcare services, reflecting the current situation where people living with dementia have varying engagement with different healthcare services. The focus was on primary care, but, where other services were specifically discussed, this was clarified.

#### Proactive care at the onset of COVID-19.

The most commonly described remote healthcare encounters by participants were telephone calls from a variety of healthcare professionals to enquire how the person living with dementia and their family were managing; these were appreciated by those who received them. Participants recalled that most of the calls were short, and did not have any particular content outside of briefly speaking to the person living with dementia or the carer:

‘ *Well, I didn’t talk very long. She* [GP] *just wanted to know I was all right and everything was OK. You know, it’s only a short call.’*

(Person living with dementia [P] 6)

Telephone calls from GPs were more often reported as occurring in the earlier months of national lockdown restrictions and were not regular calls. Instead, these calls corresponded to appointments cancelled as a result of COVID-19 restrictions.

Some people living with dementia and their carers received additional telephone calls from NHS Memory Services staff (part of secondary care services), which they had been attending. These were similarly described as mostly opportunities to ‘checkup’ on the person living with dementia:

*‘The Memory Clinic* [nurses] *do ring my sister-in-law once a week just to check-up on how Dad’s going* […] *Just to see if there’s anything he needs or how he’s doing and stuff like that.’*

(Carer [C] 12)

Carers and people living with dementia felt reassured that a healthcare professional was contacting them, particularly if they provided a contact number for future reference. In general, professionals would mostly talk to the carer, although in some calls they also spoke to the person living with dementia. Whether this was an individual conversation or not varied with regards to the carer dynamic of the dyad, but the people living with dementia that did not take part mentioned trusting their carer to manage these conversations.

Some participants who had not been contacted by any healthcare professionals during the lockdown period expressed dismay in their tone when reporting that they had not received any word from the professionals who would normally be engaging with them:

*‘I can honestly say that nobody’s checked on Mum from a medical point of view.’*

(C9)

The benefit for participants who did receive calls centred on feeling there was someone they could reach out to, which seemed to be partly owing to the content of the calls being described as not very specific.

None of the participants with dementia or a carer recalled being given any particular advice in these check-up calls, although some carers thought this could have been helpful:

*‘It would have been useful to have some advice for our family what practically can we do instead to make sure that he* [person living with dementia] *had his exercise. And to give him as much social stimulation as possible, people who understand his condition and therefore what is possible and not possible for somebody with his condition. I think that would have been very valuable indeed. We’ve tried to do our best, but I don’t think it’s been good.’*

(C21)

#### Avoidance of healthcare settings and services.

Owing to lockdown measures that mandated no unnecessary leaving of the home, social distancing, and shielding for vulnerable individuals, participants thought it was better to avoid physically visiting healthcare services.

Carers based decisions to stay away on the basis of risk of coronavirus transmission, which for them outweighed the possible benefits of the person living with dementia seeking immediate healthcare treatment, specifically in the early stages of restrictions:

*‘They said* […] *your mother will have to come here, and I wasn’t prepared to do that because I just thought it was unsafe. She was shielding, she’s been quite frail.’*

(C3)

Public Health England encouraged individuals with symptoms of COVID-19 to stay away from NHS services, self-isolate, and contact the emergency services only if needed so as to not spread the virus.^30^ This message was widely reported in the media, and demand for NHS contacts declined following the introduction of the first national lockdown measures.^31^ Participants recounted this message of patient avoidance:

*‘I mean, let’s face it, if you rang the doctors, they just said don’t come anywhere near us.’*

(C16)

*‘They don’t want me to go there, not only me, everybody.’*

(P25)

Not overburdening the NHS was another reason mentioned by some people living with dementia and their carers who did not feel they should contact or ‘trouble’ their doctor:

*‘No, not at all. I don’t want to trouble them* [GP]. *I’ve been locked in for five months. I try not to trouble anybody. They’re all very busy and it’s not fair.’*

(P27)

Some people living with dementia and their carers were managing existing problems by themselves (for example, dermatological, nutritional, or musculoskeletal problems). They had had no contact with healthcare professionals because they had not sought it out, or had no reason to:

*‘So there’s been nothing really happened during this lockdown period that we’ve had cause to really miss out on seeing the doctor. Nothing’s really happened to make us miss out.’*

(C8)

While not common, some did not seek contact because (primarily older) carers did not wish to engage with primary care by email or telephone, which was encouraged in most primary care practices (for example, e-consultations or online booking), owing to their impersonal nature and technological barriers:

*‘During the coronavirus, I’m keeping well away from them because there’s no face-to-face, it’s a lot of technology fiddling.’*

(C22)

For those who had no contact, and were not seeking any contact, carers reported providing increased care for their relative. This was owing to not only their avoidance of healthcare services as mentioned above, but also their general response to managing during the pandemic:

*‘I’ve just managed the best I can on my own.’*

(C26)

#### Difficulties with remote healthcare consultations.

During the period of lockdown and other social restrictions, healthcare provider consultations for the participants in this study were mostly by telephone. Barriers included having problems with hearing or memory problems, in which case the conversation would often be organised and handled by the carer. However, some people living with dementia who participated in remote healthcare consultations said they were content with the process and able to get what they wanted from the consultation:

*‘It’s quite all right, you know. I don’t see anything wrong with it* [telephone consultations]. *’*

(P12)

Both people living with dementia and their carers described preferring face-to-face consultations to telephone calls, because it gave them more confidence to voice their thoughts more clearly. None of the sample had experienced video consultations, although it was unclear if this was offered and refused or not offered in the first instance. Carers felt that healthcare professionals would be able to notice changes better in person, especially for what otherwise might not be addressed, and also that face-to-face encounters would enable them to discuss matters in greater depth:

*‘You don’t necessarily remember all the things you want to say over the phone whereas if you’re in the doctor’s somehow you feel more prompted to discuss things in more detail.’*

(C6)

*‘I suppose I did speak to the doctor and I was understood but you just don’t feel right by just talking to them on the phone.’*

(P6)

Some of this reluctance for remote consultations seemed to specifically relate to emerging or increasing physical problems that were more difficult to convey in a telephone conversation. Despite these limitations, some people living with dementia expressed an understanding of why remote consultations were currently needed, although this was clearer in interviews with carers:

*‘Obviously if you see somebody face-to-face, it’s much more satisfying and efficient than doing it by phone or by email or by photo. But I understand the situation why this has got to happen.’*

(C7)

Carers discussed how planning telephone consultations could be difficult, as doctors did not always keep appointments, and either phoned at different times than agreed or did not call at all. This was disruptive, and it was often up to the carer to rearrange. Telephone appointments that were not kept by professionals proved to be especially inconvenient for carers who did not live with the person with dementia. This was the case mostly for carers who were caring for a parent with dementia, as they would often be making time to support the person living with dementia:

*‘I wasn’t there for that, they* [GP] *were meant to ring up one day and I made myself available, but they didn’t ring up that day they rang up a day later.’*

(C19)

While some people living with dementia managed these consultations on their own, those adult child carers who were most affected by this reported that it could delay satisfactory resolution of problems. This affected individuals with more moderate or severe memory problems, who would either not remember the specific problem to discuss with the professional or were unable to relay the consultation to their carer.

Digital barriers or exclusion from accessing health care remotely were most often reported by older people, whether they were older (often spousal) carers or people living with dementia. To overcome this digital barrier, whether making an appointment online or working through an automated telephone attendant, they would ask for help from their children, if these were available. This was reflected by adult child carers generally not describing similar technological difficulties, some of whom voiced a preference for online consultations:

*‘I’m finding that better, because it gives you time. You can type what you want to say and then you can go back and tweak it. So, from my point of view, e-consults are easier.’*

(C11)

Although they may not prefer it, one benefit people living with dementia and carers reported was shorter waiting times and quicker access, which was associated with telephone consultations:

*P8 I don’t think it’s better, no, I’d prefer to* see *the doctor obviously, but having said that, it’s great to be able to get in the phone and talk to someone, that being the doctor.*

*C8 Without waiting a long time?*

*P8 Yes.*

This study’s sample was diverse with regards to dementia diagnosis, ethnicity, and time since diagnosis, and no clear influences on the difficulties with remote consultations were noted with regards to these characteristics.

## DISCUSSION

### Summary

This qualitative study of the experiences of remote health care during the first part of the COVID-19 pandemic shows how people living with dementia and their carers have responded, and in some cases adapted, to change.

People living with dementia and their carers reported that COVID-19 check-up calls were made at the start of the pandemic, which felt reassuring but lacked purpose and clarity. Carers felt reassured to know there was someone they could contact, but some thought this had been a missed opportunity to provide tailored advice to the person living with dementia, which would have been helpful to mitigate any potential detrimental effects of lockdown.

Various explanations were offered as to why some people living with dementia had avoided services. Carers feared the person they were caring for might catch the virus if physically visiting healthcare settings, and many were closed to patients. Government messaging further dissuaded people from visiting, and for some this also made them refrain from contacting professionals. Explanations included not wanting to overburden the NHS, as well as technology avoidance by older people living with dementia and carers, as this was not their preferred method of engaging with professionals. The study sample reflected a variety of experiences, including some people who felt they had no reason to contact professionals.

Both people living with dementia and their carers who had experienced remote consultations (typically telephone calls organised and led by the carer) found that this met their immediate needs. However, the quality of the consultation was affected: this was noted particularly for addressing new physical health needs and in remembering details of their problem without the visual cues of a face-to-face consultation. This study also highlights the potential access difficulties faced by people living with dementia without a co-resident carer. Where memory difficulties impacted on healthcare engagement, it took more effort for carers — if they were available — to maintain involvement with professionals.

### Strengths and limitations

This study is to the authors’ knowledge the first to explore the experiences of remote primary care consultations by people with dementia and their carers during the COVID-19 pandemic. Other strengths include a diverse sample with regards to ethnicity, carer relationship, accommodation status of the person living with dementia, deprivation scores, and dementia diagnoses. While the study aimed to sample purposively for variation in rurality, the sample did not reflect this, likely owing to limitations to recruitment that arose as a result of the ongoing pandemic. However, the study sample is reflective of the English population, the majority of which does live in urban areas. The multidisciplinary research team contributed to the development of the themes, contributing expertise in primary care, social care, psychology, healthcare services, ageing, and dementia. This strengthens the representativeness of the findings and highlights the potential context of relevant factors that impact on healthcare engagement. Although recruitment allowed for different types of carers to participate, it is likely that people living with dementia with no carer at all are underrepresented in this sample, and that their needs for remote healthcare consultations have not been heard. Dyadic interviews were offered if both participants agreed, and in most dyadic interviews participants were supportive and encouraging of the other sharing their point of view. On the occasions where this was more difficult, the option of individual interviews following the dyadic interview was taken up to allow for more individual in-depth discussion. However, participants who have difficulty speaking or understanding English or those with difficulty communicating through telephone or video calls are likely to be underrepresented in the study sample, although it was ethnically diverse. As part of the study inclusion criteria, participants needed to have capacity to consent, and the median year of diagnosis (2019) was relatively recent. Therefore, the findings of this study may not be applicable to those with more advanced dementia or lacking capacity. Individuals with advanced dementia are more vulnerable, especially during the COVID-19 pandemic,^32^ and they may experience greater difficulties accessing health care remotely that are not reflected in the study findings. Individuals who were not comfortable taking part in a remote interview via telephone may experience problems with remote consultations that are not included here.

### Comparison with existing literature

COVID-19-related social care service closures, such as day centres, have negatively impacted people living with dementia and their carers,^33^ which makes the continuation of high-quality primary care even more important. Concerns have been raised about increasing loneliness, low mood, and deconditioning of older people related to prolonged isolation and lack of exercise following lockdown.^34^ The lack of structure and content of proactive check-up calls found in this study is a missed opportunity for providing tailored advice to mitigate this. Research regarding the impact of COVID-19 on remote healthcare consultation is emerging, and initial quantitative findings describe the high uptake of remote consultations, specifically also for frail older adults.^35^ The findings presented in this paper provide a deeper understanding of both the experiences of those engaging in remote consultations, as well as the reasons why individuals may not seek contact with healthcare professionals, which have otherwise been speculative.^36^

To provide person-centred care, it is important to engage with the person living with dementia in order to include their voice and perspective alongside that of their carer,^37^ and this may require more adjustments during remote consultations. This should be extended to include choices regarding the method of remote consultation, as this can be an opportunity for those involved to contribute to care decisions to the best of their abilities.

### Implications for research and practice

Practices need to be aware that, although remote healthcare consultations are feasible, people living with dementia and carers prefer face-to-face contact. Issues may arise in particular when trying to describe new problems over the phone, and practice staff should consider whether it is then more appropriate to see people in person. Practices should ensure that proactive calls are opportunities to discuss managing well during periods of social restriction with both the person living with dementia and their carer. Future implementation of check-up calls specifically could provide opportunities for tailored advice on how to maintain wellbeing, with regards to physical exercise, contingency planning, or social engagement.

Language difficulties or missed visual cues may make it more difficult for those living with dementia to describe their problems in full remotely. Primary care practitioners should consider ways to mitigate this, for example, by advising patients living with dementia or carers to prepare a checklist of what to cover in a telephone encounter in advance, or allowing for more extended phone calls with time to prompt further and explore concerns. There may also be potential to involve other family members to provide opportunities for video consultations if digital literacy is a barrier, which was a technique used by some of participants in this study to overcome similar technological problems. Participants had been contacted by primary and secondary care professionals, and they reported similar experiences for both, highlighting areas of coordination, record keeping, and improvement for future initiatives across services.

Future research should explore the attitudes of other parts of the patient population, specifically those who have experienced or were offered video consultations, as this was not recounted by this sample. Other vulnerable populations, such as those living in care homes or with increased digital barriers, should be encouraged to be a focus of future research. The views and experiences of GPs providing remote consultations during the COVID-19 pandemic should be explored in depth, especially regarding encounters with their patients who may have reduced cognitive capacity. Future longitudinal analysis regarding remote consultations and the development of strategies to maintain wellbeing as the pandemic continues may provide further insight into good practice for primary care professionals engaging with people living with dementia and their carers.
